# The effect of heavy metals on the viability of *Tetraselmis marina* AC16-MESO and an evaluation of the potential use of this microalga in bioremediation

**DOI:** 10.7717/peerj.5295

**Published:** 2018-07-25

**Authors:** Henry Cameron, Maria Teresa Mata, Carlos Riquelme

**Affiliations:** Centro de Bioinnovación Antofagasta (CBIA), Faculty of Marine Sciences and Biological Resources, Antofagasta University, Antofagasta, Chile

**Keywords:** Microalgae, Tetraselmis, Heavy metals, Bioremediation, Bioremediation

## Abstract

The use of microalgae in biotechnological processes has received much attention worldwide. This is primarily due to the fact that they are inexpensive to grow, requiring only sunlight and CO_2_, whilst lending themselves to a range of uses, such as to reduce CO_2_ levels, as fish feed, in biofuel production, for the generation of secondary metabolites of interest, and in bioremediation. These features mean that microalgae are excellent candidates for the implementation of a range of eco-friendly technologies. Here, we investigated the behavior and feasibility of the use of the microalgal strain *Tetraselmis marina* AC16-MESO against heavy metal contamination focused on potential use in bioremediation. The following key parameters were recorded: (i) the sedimentation efficiency, which reached 95.6% after five hours of decantation; (ii) the ion tolerance (Ca^2+^, Co^2+^, Cu^2+^, Fe^3+^, Mn^2+^ and Ni^2+^) at concentrations of 0.1, 1.0, 5.0, 10.0 and 20.0 mg*L^−1^ and (iii) ion removal efficiency (Cu^2+^, Fe^3+^ and Mn^2+^). Our results indicated a higher tolerance for iron and calcium (20 ± 1.10 mg*L^−1^; 100 ± 8.10 mg*L^−1^), partial to nickel, manganese and copper (4.4 ± 0.10 mg*L^−1^; 4.4 ± 0.15 mg*L^−1^; 5 ± 1.25 mg*L^−1^) and less for cobalt (0.1 ± 0.20 mg*L^−1^). Moreover, removal efficiency of 40–90% for Cu^2+^, 100% for Fe^3+^, and 20–50% for Mn^2+^ over a 72 hours period, for ion concentrations of 1.0 and 5.0 mg*L^−1^.

## Introduction

Every day, industrial processes generate large amounts of contaminated water, which are discharged into the environment. Most pollution in these waters is due to heavy metals (HMs) such as copper, chromium, nickel, iron, cadmium, and arsenic (Doshi et al., 2008). Due to their non-biodegradability and hazardous characteristics, heavy metals pose a great threat to the health of the environment. Aquatic organisms acquire heavy metals directly from contaminated water or through the food chain. Prolonged exposure of soils to heavy metals may result in a marked decrease in soil enzyme activities (Irha, Slet & Petersell, 2003). It is therefore of utmost importance to remove HMs from the industrial wastewaters released into water courses and soil (Doshi et al., 2008).

Current conventional methods for the removal of metals from industrial wastewater include chemical precipitation, ion exchange and membrane purification (Chen et al., 2002). However, these conventional approaches are often ineffective or expensive, especially when the metals in solution are in the range of 1–100 mgL^−1^ (Kumar et al., 2015). In addition, some of these methods have the disadvantage of producing toxic sludge (Volesky, 2001; Ahalya, Ramachandra & Kanamadi, 2003; Ahluwalia & Goyal, 2007). The necessary treatment of this sludge requires large amounts of energy and chemical reagents (Ahalya, Ramachandra & Kanamadi, 2003). There is therefore great interest in the development of innovative, cost-effective, efficient and sustainable methods for the removal of toxic substances from wastewater and aquatic ecosystems. Recent years have seen the development of unconventional technologies for the prevention of HM contamination, the decrease of HM concentrations (e.g., by reducing the flow of HMs into water courses and soil), and for the removal of HMs from the contaminated milieu via remediation (Kumar et al., 2015). Among these techniques, bioremediation shows great potential to make an eco-friendly contribution to HM decontamination. In addition, it may facilitate at least a part recovery of certain metals of interest (Wilde & Benemann, 1993).

The advantages of microalgae for the biosorption of HMs have widely been recognized (Kumar et al., 2015). The effectiveness of marine algae in the remediation of metal ions has been demonstrated (Volesky, 1990), and microalgal biomass has been shown to capture different metals, with different species showing an affinity to different metal ions (Doshi et al., 2008). These algae are therefore a promising candidate for use as a biosorbent material for the low-cost removal of contaminants. Marine and freshwater algae have already been used in adsorption and elution of gold, silver and cobalt (Hamdy, 2000; Fujita, Kuzuno & Mamiya, 1992). Furthermore, the efficiency of certain algae in HMs removal has been reported to be greater than that of activated coal, natural zeolites and synthetic ion-exchange polymers (Volesky, 1992). The kinetics of HM removal by biologically active microalgae can be divided in two steps: an initial phase of physical adsorption to the cell surface, and a second, slow, phase called biosorption, which relies on intracellular transport and chelation (Folgar et al., 2009; Nourbakhsh et al., 1994). Adsorption hence relies on various processes, including ion exchange, metal ion chelation and micro-precipitation, all of which occur at the cell wall. Differences in these processes among microalgae result in different remediation efficiencies (Zhao et al., 2013).

On this background, the behavior and feasibility of *Tetraselmis marina* AC16-MESO was assessed directed at water treatment isolated off the coast of Antofagasta, Chile. Key parameters such as sedimentation efficiency, cell viability and HMs removal capacity was evaluated.

## Materials and Methods

### Microalgae

The following microalgae were used: *Muriellopsis* sp*., Nannochloropsis gaditana* and *Tetraselmis marina* AC16-MESO. *Tetraselmis* was isolated from the intertidal area of the San Jorge Bay, Antofagasta, specifically from the area known as the Beach of the Oil Companies (”Playa de las Petroleras”), and identified and preserved at the University of Antofagasta marine mesocosm facility (Mata et al. in press). Algae were grown in Erlenmeyer flasks of 2 liters until reaching sufficient biomass for analysis, in UMA5 medium (Riveros et al., 2018) (NaNO_3_ 4.55*10^−5^ M; NaH_2_PO_4_*H_2_O 2.41*10^−4^ M; NaHCO_3_ 1.99*10^−3^ M) at 20 °C and a continuous photosynthetic photon flux of 70 µmol m^−2^s^−1^(24 h light).

### Sedimentation efficiency

In order to evaluate the potential use of *Tetraselmis marina* AC16-MESO in bioremediation, sedimentation efficiency was determined and subsequently compared it to that of the microalgae *Muriellopsis* sp. and *Nannochloropsis gaditana*, which are characterized by high and low sedimentation efficiencies (SE), respectively. Samples of the microalgal suspension were taken and diluted in a cuvette of polystyrene (Sartedt, Nümbrecht, Germany), the suspension was left to settle at 22 °C in the dark in a spectrophotometer (Pharo 300; Merck, Kenilworth, NJ, USA). During the settling period, turbidity of the sample was measured at 550 nm at the same height in the cuvette to determine the sedimentation activity.

Sedimentation efficiency (SE) was calculated according to Eq. (1) (Smith & Davis, 2012):

(1) \begin{eqnarray*}SE=1-A/{A}_{0}\end{eqnarray*}

where *A* corresponds to the supernatant’s absorbance at 550 nm at time *t* (120 min), and A_0_ corresponds to the absorbance of the initial suspension culture.

### Heavy metal assays

Stock solutions of each ion (Ca^2+^, Co^2+^, Cu^2+^, Fe^3+^, Mn^2+^ and Ni^2+^) were prepared at a concentration of 50 mg*L^−1^ in distilled water, using the following salts: calcium nitrate tetrahydrate (Ca(NO_3_)_2_*4H_2_O), Cobalt (II) chloride hexahydrate (CoCl_2_*6H_2_O), copper (II) sulfate pentahydrate (CuSO_4_*5H_2_O), iron (III) chloride hexahydrate (FeCl_3_*6H_2_O), manganese (II) chloride tetrahydrate (MnCl_2_*4H_2_O) and nickel (II) sulfate hexahydrate (NiSO_4_*6H_2_O). All the glass and plastic material was washed with a solution of 10% (v/v) hydrochloric acid for 12 h and rinsed with distilled water in order to remove any contamination prior to use.

#### Toxicity assay (EC_50_)

The effect of Ca^2+^, Co^2+^, Cu^2+^, Fe^3+^, Mn^2+^ and Ni^2+^ ions on the microalga *Tetraselmis marina* AC16-MESO was studied in an EC_50_ assay, which measures concentrations resulting in a 50% of maximal effect of the test organisms (Hu, Luo & Huang, 2014). All assays were carried out in 96-well plates with an initial inoculum of 2 ×10^5^ cells*mL^−1^ in 200 µL of marine saline solution (7 mg*L^−1^ MgSO_4_*7H_2_O; 0.8 mg*L^−1^ KCl; 24 mg*L^−1^ NaCl) autoclaved at 121 °C. Increasing metal concentrations were added (0.001, 0.01, 0.1, 1, 5, 10; 20; 40; 80; 100 mg/L). Conditions were controlled throughout, with a temperature of 20 °C and continuous illumination at 70 µmol m^−2^s^−1^. The respective heavy metal treatments were applied in triplicate for 72 h. Cell toxicity was assessed by tracking the OD_550_ as a proxy for the number of cells. The linear relationship between microalgal density and OD_550_ is shown in Eq. (2) of Toxicity assay EC_50_. All the glass and plastic material was washed with a solution of 10% (v/v) hydrochloric acid for 12 h and rinsed with distilled water in order to remove any contamination prior to use.

#### Effect of metal ions on *Tetraselmis marina* AC16-MESO

For each metal, cellular viability was evaluated by tracking cell density at 0, 24, 48, 72 and 96 h at the following metal concentrations (in mgl*L^−1^): 0.0 (control), 0.1, 1.0, 5.0, 10.0, and 20.0. All assays were conducted in triplicate. The sample of precultivated microalgae was centrifuged at 6,000 rpm for 10 min, and the supernatant was discarded. The pelleted microalgal cells were washed twice with sterile Milli-Q water to remove impurities and re-suspended in sterile Milli-Q water for inoculation into the growth medium. Microalgae were added to flasks containing 250 mL modified f/2 medium prepared with artificial seawater, with absence of trace elements (Goldman & McCarthy, 1978), at a concentration of 2 ×10^5^ cells*mL^−1^. Each metal was added separately to achieve the concentrations described above. Microalgal cultures were kept at 20 °C under continuous exposure to light (70 µmol m^−2^s^−1^) and constant aeration.

The cell density of the microalgal suspension was tracked by daily optical density (OD) measurements at 550 nm (Zhou et al., 2012). Measurements were carried out in 96-well plates using a microplate reader (GloMax Multi Detection System; Promega, Madison, WI, USA). The linear relationship between microalgal density and OD_550_ is given by Eq. (2).

(2) \begin{eqnarray*}\text{Microalgal density}=1,050,153.16\;{\mathrm{OD}}_{550}+3,483.29({R}^{2}=0.99).\end{eqnarray*}

#### Heavy metal removal capacity

To evaluate the metal removal capacity, they selected the ions whit best results for cellular growth and toxicity assay (Cu^+2^, Mn^+2^ and Fe^+3^). microalgae were added to one liter of UMA 5 medium prepared with artificial seawater with absence of trace elements (Goldman & McCarthy, 1978) at a concentration of 2 ×10^5^ cells*mL^−1^. Each metal was added at final concentrations of 1 and 5 mg*L^−1^. Metal ion concentrations in the culture medium were checked after 72 h using a colorimetric kit (Spectroqant^®^ Merck) and a spectrophotometer (Pharo 300; Merck, Kenilworth, NJ, USA). 50 mL samples were taken from the culture, and after leaving the microalgae to settle, the remaining metal ion concentration in the supernatant was measured. Pure medium was used as a blank. All assays were performed in triplicate. Lighting and temperature remained constant throughout at 72 µmol m^−2^s^−1^ and 20 ± 1 °C, respectively.

### Statistical analyses

All assays were performed in triplicate. EC_50_, effect of metal ions on cell density and HM removal assays were evaluated using a Bonferroni-corrected by one way-ANOVA at a statistical significance threshold of *p* ≤ 0.05, using the software GraphPad Prism version 5.01.

## Results

### Sedimentation efficiency

**Figure 1:**
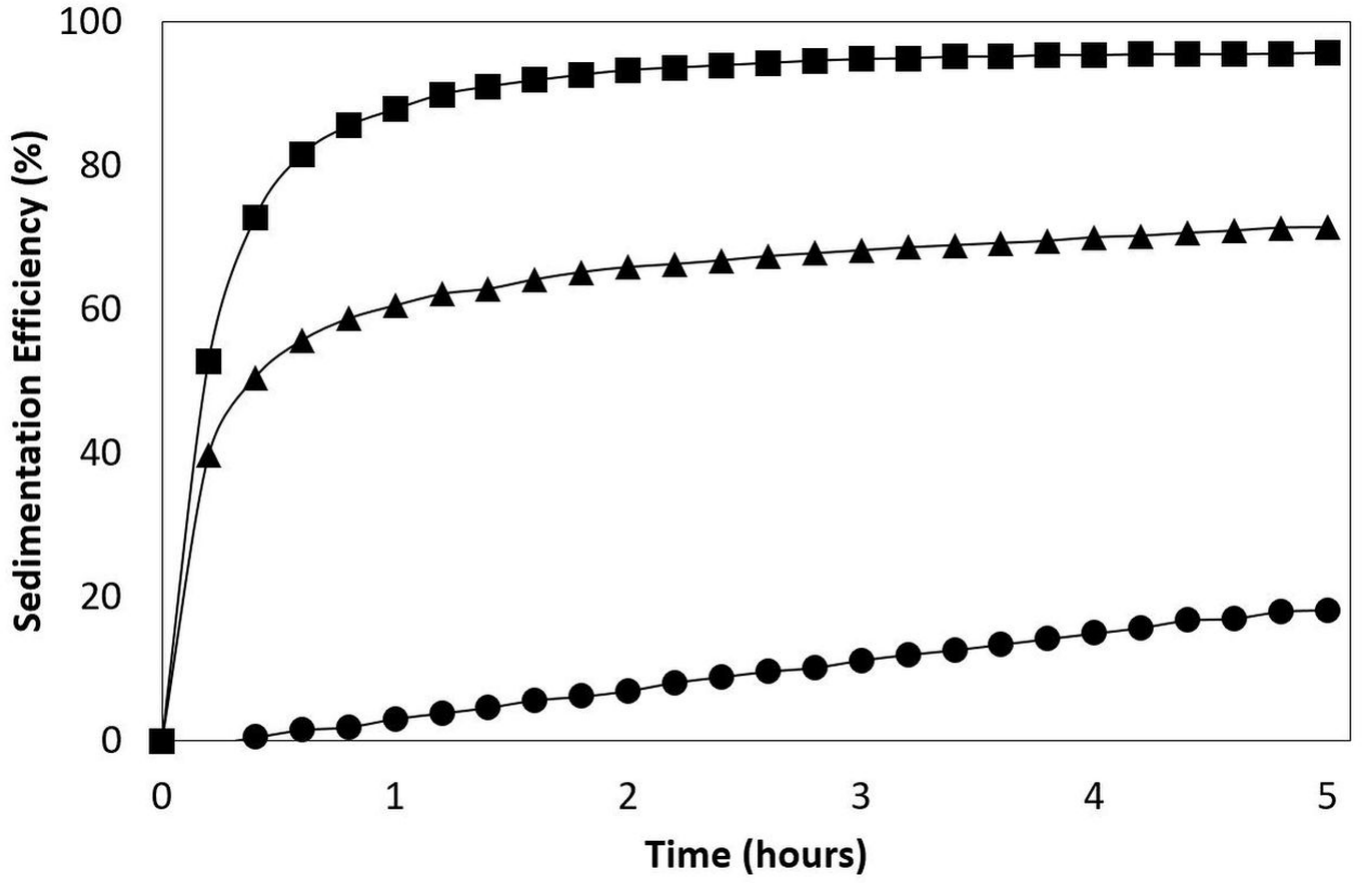
Sedimentation efficiency (SE). *Tetraselmis marina* AC16-MESO (■) and the reference microalgaes *Muriellopsis* sp. (▴) and *Nannochloropsis gaditana* (• ). LTIT 1.

Different, microalgae were studied; the freshwater microalgae *Muriellopsis sp.;* marines microalgae such as *T.* AC16-MESO and *N. gaditana.* The SE of the flocculating microalgae were higher than those of non-flocculant microalgae. For example, *T.* AC16-MESO reached 95,6%, above *Muriellopsis sp*. (71,3%), and substantially over that of *N. gaditana* (18,2%), measured at 5 h (Fig. 1).

### Acute toxicity (EC_50_)

**Figure 2:**
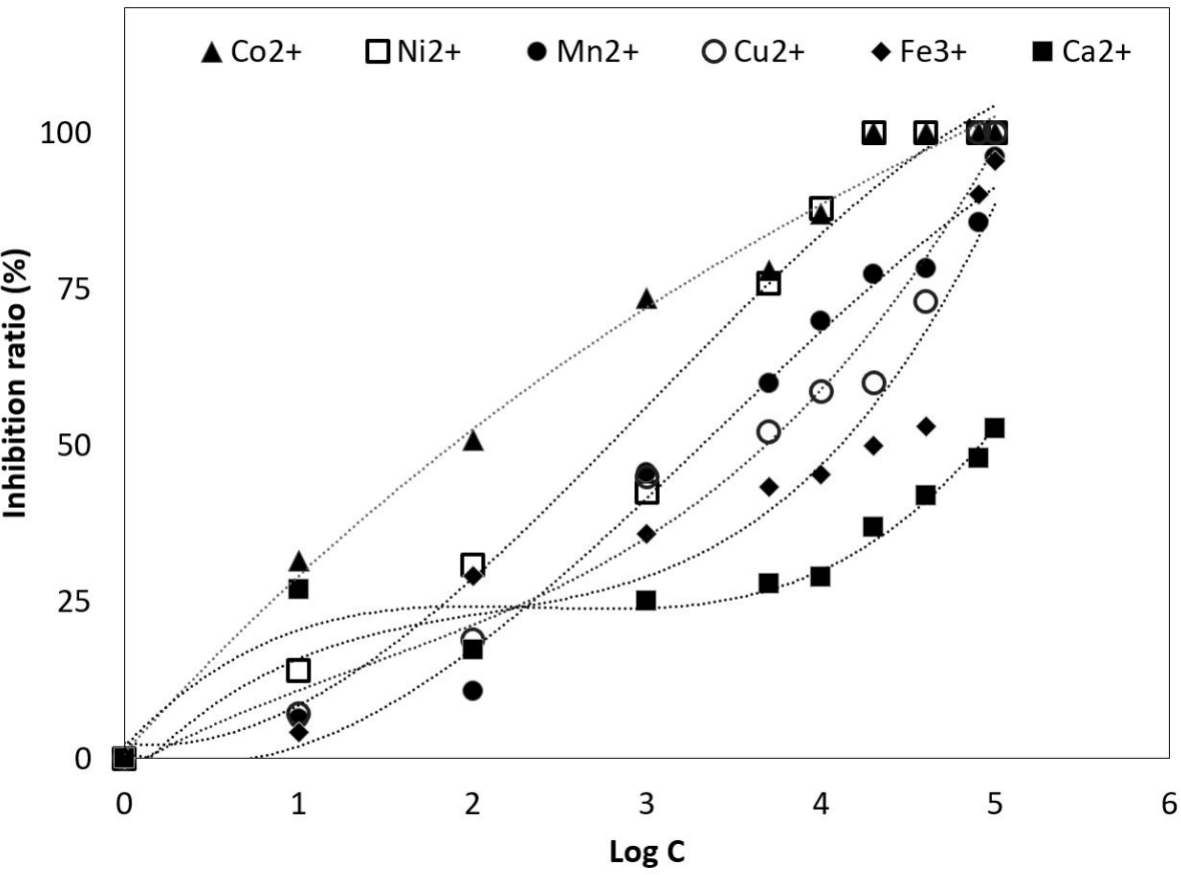
Half maximal effective concentration (EC_50_.) *Tetraselmis marina* AC16-MESO at different concentration of metal ions. (▴) Co^2+^; (□) Ni^+2^; (•) Mn^2+^; ($○ $) Cu^2+^; (♦) Fe^3+^ and (■) Ca^2+^. The *y*-axis shows the inhibition ratio (%) for each ion at concentrations of 0.0 (control), 0.001, 0.01, 0.1, 1, 5, 10, 20, 40, 80 and 100 mg*L^−1^. The EC_50_ was determined for each ion.

In Fig. 2 shows the curves of EC_50_ obtained for each of the ions and concentrations tested. Co^2+^ ion was the lowest concentration tested, reaching a value of 0.1 ± 0.20 mg*L^−1^after 72 h. On the other hand, Ni^2+^; Mn^2+^ and Cu^2+^ ions were better tolerated, with EC_50_ values of 4.4 ± 0.10 mg*L^−1^; 4.4 ± 0.15 mg*L^−1^ and 5 ± 1.25 mg*L^−1^. By contrast, EC_50_ values for Fe^3+^ and Ca^2+^ ions reached higher tolerance, reaching values of 20 ± 1.10 mg*L^−1^ and 100 ± 8.10 mg*L^−1^.

### Effect of metal ions on cell density of *Tetraselmis marina* AC16-MESO

**Figure 3:**
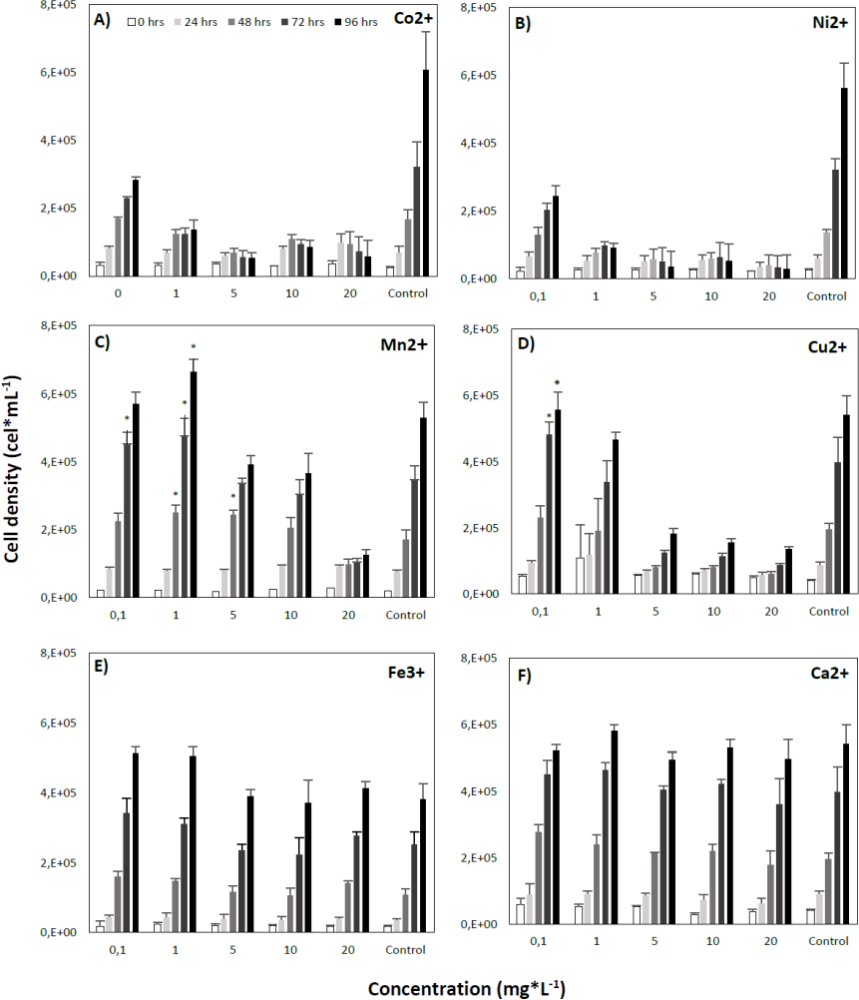
Effect of different concentrations of metal ions on the growth of the microalga *Tetraselmis marina* AC16-MESO. (A) Co^2+^; (B) Ni^+2^; (C) Mn^2+^; (D) Cu ^2+^; (E) Fe^3+^ and (F) Ca^2+^. For each metal, the microalga was cultured at ion concentrations of 0.0 (control), 0.1, 1.0, 5.0, 10.0 and 20.0 mg*L^−1^, and cellular densities were measured at 0, 24, 48, 72 and 96 h. Asterisks indicate significant differences between metal treated and control cultures at each time of measurement, at a 95% confidence level after a Bonferroni correction for *n* = 3, at *p* < 0.05. All assays were carried out in triplicate.

The lowest concentrations of Co2+ ions significantly decreased cell density values below those of the control. After 96 h, cell densities were substantially lower than in the control, by as much as 322,759 (53%) and 548,874 (90%) cells*mL^−1^ at concentrations of 0.1 and 20.0 mg*L- 1, respectively (Fig. 3A). Likewise, cultivation with Ni2+ at any concentration led to a significant decrease in cell densities compared to the control (Fig. 3B).

On the other hand, even at low concentrations of Mn2+, a significant increase in cell density over control values was observed. After 72 h at a concentration of 0.1 mg*L^−1^, cell densities exceeded those of the control by 105,008 (30%) cells*mL- 1, and after 48, 72 and 96 h at a concentration of 1 mg*L^−1^, control cell densities were exceeded by 79,515 (46%), 130,029 (38%) and 133,611 (25%) cells*mL^−1^, respectively. Higher concentrations led to a decrease of cell density relative to the control (Fig. 3C).

By contrast, in the presence of Cu2+, the microalgal suspension reached significantly higher cell densities than the control. Control cell densities were exceeded by 83,794 (21%) and 14.212 (3%) cells*mL^−1^ at a concentration of 0.1 mg*L^−1^ after 72 and 96 h of cultivation, respectively. However, at higher Cu2+ concentrations, a decrease of cell density was observed, and after 96 h of cultivation, final cell densities were 359,280 (66%), 384,935 (71%) and 406,170 (75%) cells*mL^−1^ below those of the control, at concentrations of 5.0, 10.0 and 20.0 mg*L^−1^, respectively (Fig. 3D).

Fe3+ also led to a significant increase in cell density compared to the control, and at a concentration of 0.1 mg*L^−1^, cell density values exceeded those of the control by 51,691 (47%), 89,402 (35%) and 132,243 (34%) cells*mL^−1^ after 48, 72 and 96 h, respectively. At a concentration of 1.0 mg*L- 1, cell density values exceeded those of the control by 57,191 (23%) and 124,663 (33%) cells*mL- 1 after 72 and 96 h, respectively. At the remaining concentrations, no significant differences in cell density compared to the control were seen (Fig. 3E). Finally, Ca2+ did not result in a significant variation of microalgal cell density over the sampling period, nor there were any significant differences in cell density with respect to the control at the different concentrations employed (Fig. 3F).

### Heavy metal removal

**Figure 4:**
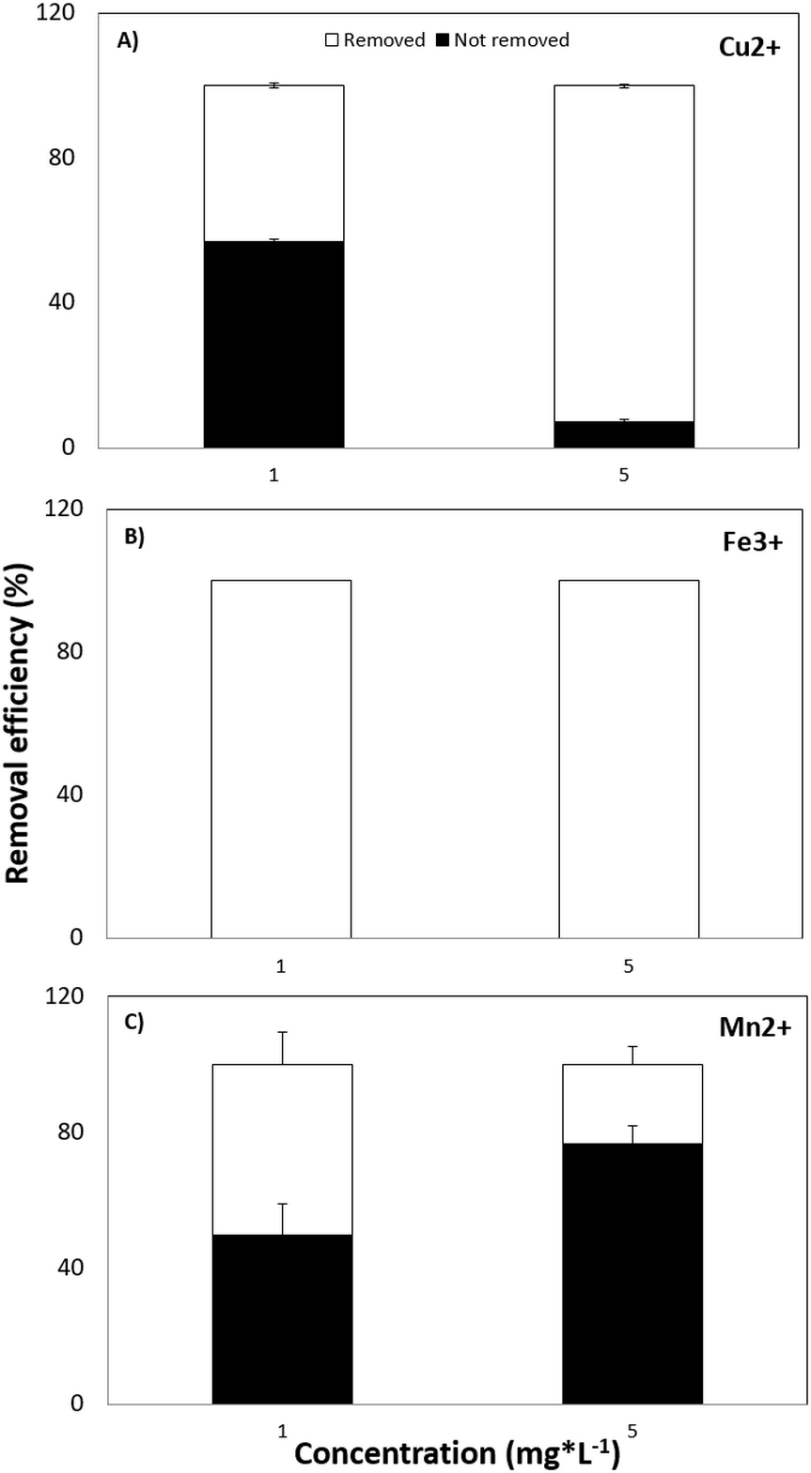
Removal efficiency of MPS. (A) Cu^2+^, (B) Fe^3+^ and (C) Mn^2+^ by *Tetraselmis marina* AC16-MESO. The *y*-axis shows the removal efficiency (%) at 72 h, at ion concentrations of 1.0 and 5.0 mg*L^−1^.

Finally, the capacity of *Tetraselmis marina* AC16-MESO to remove those ions who had given the best results in the cell density assays while resulting least toxic in EC_50_ assays over 72 h. The efficiency of Cu^2+^, Fe^3+^ and Mn^2+^ removal at concentrations of 1.0 and 5.0 mg*L^−1^ was tested. For Cu^2+^, the removal percentage was 42.9% at 1.0 mg*L^−1^ and 92% at 5.0 mg*L^−1^ (Fig. 4A). For Fe^3+^, it was 100%, both at 1.0 and 5.0 mg*L^−1^ (Fig. 4B). Finally, for Mn^2+^, the removal percentage was 50.4% at 1.0 mg*L^−1^ and 23.4% at 5.0 mg*L^−1^ (Fig. 4C).

## Discussion

### Sedimentation efficiency

Microalgae are considered a workable alternative for the remediation of heavy-metal contaminated environments. However, the collection of biomass from microalgal cultures represents a significant hurdle for the economically viable development of this process (Alam et al., 2015). Harvesting in commercial microalgae production plants in generally done by centrifugation. Different studies showed a contribution of the costs for harvesting to more than 30% of the total cost in case of algal production in open ponds (Zittelli et al., 2006). The microalga used here, *Tetraselmis marina* AC16-MESO, is autoflocculating; its biomass could therefore efficiently be harvested via sedimentation at a very minor cost. Compared to two microalgal reference strains, one with a high sedimentation efficiency, the other with a low one, *Tetraselmis marina* AC16-MESO showed a good capacity sedimentation efficiency of 95,6% for 5 h, in contrast to *Muriellopsis sp.* (71,3%) and *Nannochloropsis gaditana* (18,2%). The separation of *Tetraselmis marina* biomass via decantation is therefore practicable and could be achieved in a relatively short time, and at low cost.

### Effect of heavy metal ions on the growth of *Tetraselmis marina* AC16-MESO

Co2+ and Ni2+ both had an inhibitory effect on the cellular growth of *Tetraselmis marina* AC16-MESO at all concentrations tested here. By contrast, low concentrations of Co2+ (0.1 and 0.5 mg*L^−1^) have been found to boost the cellular growth of the chlorophyte microalga *Monoraphidium minutum* by 8 to 13% over 240 h, while Co2+ concentrations of 0.5 and 1.5 mg*L^−1^ increased the cellular growth of *Nytzchia perminuta* by 5 to 9% (El-Sheekh et al., 2003). For Ni2+, on the other hand, the literature is consistent with the findings of the present study. The cellular growth of *Ankistrodesmus falcatus* over 96 h has been found to be inhibited by Ni2+ concentrations of 30, 60, and 120 µg*L^−1^ (Martínez-Ruiz & Martínez-Jerónimo, 2015). These concentrations are substantially below those applied in the present study. Meanwhile, a negative effect was also found in the lowest tested concentration, 0.1 mg*L^−1^. These findings show that, compared to the other ions, Ni2+ has a considerable negative effect on cellular viability.

On the other hand, Manganese is an essential cofactor in photosynthesis; its deficiency leads to the inhibition of photosystem II (Yang et al., 2015). At micro-concentrations, Mn2+ is fundamental to the optimum growth of *Dunaliella tertiolecta* (Chen et al., 2011). Yang et al. (2015) found a 6% increase of *Chlorella minutissima* biomass compared to a control after 168 h at a Mn2+ concentration of 213 mg*L^−1^. Here, increased cell density for *Tetraselmis marina* AC16-MESO at concentrations much below those applied by Yang et al. (2015), reaching a 30% increase with respect to the control after 72 h at a concentration of 0.1 mg*L^−1^, and a 38% increase at a concentration of 1.0 mg*L^−1^. Nevertheless, Concentrations above 10.0 mg*L^−1^ had a significant negative effect on microalgal growth.

By contrast, Low concentrations of copper, iron and manganese even accelerated its cellular growth. At concentrations between 0.2 and 0.5 mg*L^−1^, Cu2+ has been reported to enhance the growth of the microalgae *Chlorella pyrenoidosa* and *Scenedesmus obliquus* (Zhou et al., 2012). Here, the Cu2+ had a positive effect on the growth of *Tetraselmis marina AC16-MESO* at a concentration of 0.1 mg*L^−1^ was observed, leading to a 21% increase in cell density compared to the control over a period of 72 h. Likewise, a 14% increase in cell density has been reported for cultures of *Isochrysis galbana* at 0.6 mg*L-1 Fe3+ over 408 h (Liu & Wang, 2014). Here, a Fe3+ concentration of 0.1 mg*L^−1^ increased *Tetraselmis marina* AC16-MESO cell density by 35% over 72 h compared to the control. A Fe3+ concentration of 1.0 mg*L^−1^ led to a 23% increase.

Finally, Ca2+ has a central role in many processes related to plant development and growth (Hepler, 2005). At a concentration of 6.4 mg*L^−1^, it has been reported to boost the increase in cellular density of *Chlorella vulgaris* over 432 h by 20% compared to a control, as well that of *Scenedesmus obliquus* by 25% compared to a control, (Gorain, Bagchi & Mallick, 2013). By contrast, Ca2+ was not found to have any effect on the cellular growth of *Tetraselmis marina* AC16-MESO at any of the concentrations tested. However, the period of culture was shorter (96 h).

### Heavy metal removal

The maximal removal efficiency was 90% for Cu^2+^, 100% for Fe^+3^and 50% for Mn^2+^, all at 72 h. A copper removal efficiency of close to 100% has been described for the microalgae *Chlorella pyrenoidosa* and *Scenedesmus obliquus* (Zhou et al., 2012). While the removal of iron and copper reached a maximum during the initial adsorption phase (at 72 h), the efficiency of Mn^2+^ removal was only 50% in the same period. In this context, it is worth mentioning that no significant HM removal has been reported beyond between 96 and 120 h, indicating that at that point, the microalgal cells may have reached saturation with HMs (Alam et al., 2015). In line with this, the removal of copper ions has shown to reach a maximum in the first days of culture, with only an insignificant increase happening after that (Zhou et al., 2012). This change of efficiency over time can be explained by the complexation of metal ions by functional groups at the cell surface and the increasing competition between ions, as the availability of free complexation sites decreases within the biomass (Kumar et al., 2015).

## Conclusion

The efficiency of HM removal by microalgae depends on the microalgal species, the properties and concentration of the metal ion, and the period of culture. We found the microalga *Tetraselmis marina* AC16-MESO to tolerate, and to be capable of the removal of high concentrations of metal ions. In addition, it was capable of removing these metals at a high rate, within a relatively short time and with a high sedimentation efficiency. These characteristics make of *Tetraselmis marina* AC16-MESO a promising candidate for use in bioremediation and a model for the use of microalgae in the bioremediation of water contaminated with copper, iron and manganese.

##  Supplemental Information

10.7717/peerj.5295/supp-1 Figure S1 Sedimentation efficiency (SE) *Tetraselmis marina* AC16-MESO (■) and the reference microalgaes *Muriellopsis* sp. (▴) and *Nannochloropsis gaditana* (•).

10.7717/peerj.5295/supp-2Figure S2Half maximal effective concentration (EC_50_)*Tetraselmis marina* AC16-MESO at different concentration of metal ions. () Co^2+^; (**■**) Ni^+2^; (•) Mn^2+^; ($○ $) Cu^2+^; (♦) Fe^3+^ and (■) Ca^2+^. The y-axis shows the inhibition ratio (%) for each ion at concentrations of 0.0 (control), 0.001, 0.01, 0.1, 1, 5, 10, 20, 40, 80 and 100 mg*L^−1^. The EC_50_ was determined for each ion.

10.7717/peerj.5295/supp-3 Supplemental Information 1 Effect of metal ions on cell density of *Tetraselmis marina* AC16-MESO** Effect of different concentrations of metal ions on the growth of the microalga *Tetraselmis marina* AC16-MESO. (A) Ca^2+^; (B) Co^2+^; (C) Cu^2+^; (D) Fe^3+^; (E) Mn^2+^; and (F) Ni^2+^. For each metal, the microalga was cultured at ion concentrations of 0.0 (control), 0.1, 1.0, 5.0, 10.0 and 20.0 mg*L^−1^, and cellular densities were measured at 0, 24, 48, 72 and 96 h. Asterisks indicate significant differences between metal treated and control cultures at each time of measurement, at a 95% confidence level after a Bonferroni correction for *n* = 3, at *pλτ*0.05. All assays were carried out in triplicate.

10.7717/peerj.5295/supp-4 Supplemental Information 2 Heavy metal removal Efficiency of (A) Cu^2+^; (B) Fe^3+^ and (C) Mn^2+^ removal by *Tetraselmis marina* AC16-MESO. The y-axis shows the removal efficiency (%) at 72 h, at ion concentrations of 1.0 mg*L^−1^ and 5.0 mg*L^−1^.

## Additional Information and Declarations

### Data Availability

The following information was supplied regarding data availability: The raw data are provided in the Supplemental Files.
